# Supporting people leaving prisons during COVID-19: perspectives from peer health mentors

**DOI:** 10.1108/IJPH-09-2020-0069

**Published:** 2021-02-17

**Authors:** Katherine E. McLeod, Kelsey Timler, Mo Korchinski, Pamela Young, Tammy Milkovich, Cheri McBride, Glenn Young, William Wardell, Lara-Lisa Condello, Jane A. Buxton, Patricia A. Janssen, Ruth Elwood Martin

**Affiliations:** School of Population and Public Health, University of British Columbia, Vancouver, Canada and the Collaborating Centre for Prison Health and Education, University of British Columbia, Vancouver, Canada; Interdisciplinary Studies, University of British Columbia, Vancouver, Canada; School of Population and Public Health, University of British Columbia, Vancouver, Canada and the Collaborating Centre for Prison Health and Education, University of British Columbia, Vancouver, Canada; Justice Studies, Nicola Valley Institute of Technology, Burnaby, Canada and the Collaborating Centre for Prison Health and Education, University of British Columbia, Vancouver, Canada; School of Population and Public Health, University of British Columbia, Vancouver, Canada; the Collaborating Centre for Prison Health and Education, University of British Columbia, Vancouver, Canada and the BC Centre for Disease Control, Vancouver, Canada; School of Population and Public Health, University of British Columbia, Vancouver, Canada and BC Children's Hospital Research Institute, Vancouver, Canada; School of Population and Public Health, University of British Columbia, Vancouver, Canada and the Collaborating Centre for Prison Health and Education, University of British Columbia, Vancouver, Canada

**Keywords:** Public health, Prison, Throughcare, Qualitative research, COVID-19, Peer health mentor

## Abstract

**Purpose:**

Currently, people leaving prisons face concurrent risks from the COVID-19 pandemic and the overdose public health emergency. The closure or reduction of community services people rely on after release such as treatment centres and shelters has exacerbated the risks of poor health outcomes and harms. This paper aims to learn from peer health mentors (PHM) about changes to their work during overlapping health emergencies, as well as barriers and opportunities to support people leaving prison in this context.

**Design/methodology/approach:**

The Unlocking the Gates (UTG) Peer Health Mentoring Program supports people leaving prison in British Columbia during the first three days after release. The authors conducted two focus groups with PHM over video conference in May 2020. Focus groups were recorded and transcribed, and themes were iteratively developed using narrative thematic analysis.

**Findings:**

The findings highlighted the importance of peer health mentorship for people leaving prisons. PHM discussed increased opportunities for collaboration, ways the pandemic has changed how they are able to provide support, and how PHM are able to remain responsive and flexible to meet client needs. Additionally, PHM illuminated ways that COVID-19 has exacerbated existing barriers and identified specific actions needed to support client health, including increased housing and recovery beds, and tools for social and emotional well-being.

**Originality/value:**

This study contributes to our understanding of peer health mentorship during the COVID-19 pandemic from the perspective of mentors. PHM expertise can support release planning, improved health and well-being of people leaving prison and facilitate policy-supported pandemic responses.

## Introduction

During the current COVID-19 pandemic, people who experience incarceration face a disproportionate risk of infection and harm (Jiménez *et al.*, 2020; Kinner *et al.*, 2020). The World Health Organization and the UN Human Rights Commissioner have stressed the need for human rights-oriented public health responses in correctional settings (United Nations Office of the High Commissioner on Human Rights, 2020; World Health Organization, 2020). In Canada and other jurisdictions, one strategy to address this risk widely discussed in the early days of the pandemic is to decrease prison populations (Burki, 2020; Jiménez *et al.*, 2020; Statistics Canada, 2020). In British Columbia (BC), the population of provincial facilities declined by 26% between February and April of 2020 (Statistics Canada, 2020). However, this discussion of decarceration often does not include the health and social supports which are critically important for people being released.

Prisons concentrate populations of people who have experienced or are experiencing health and social inequities including trauma and violence, homelessness and/or vulnerable housing, poverty and harmful substance use (Western and Pettit, 2010; Kouyoumdjian *et al.*, 2016). When leaving prison people face systemic barriers which impact their health and well-being, including unmet needs for housing, health care, clothing and treatment services (Geller and Curtis, 2011; Fahmy *et al.*, 2018; McLeod *et al.*, 2020), and access to culturally safe services for Indigenous Peoples. COVID-19 has increased barriers as community resources such as treatment centres, recovery houses, harm reduction and healthcare services, indigenous friendship centers and shelters are closed or running with decreased capacity (Faculty of Arts, 2020; McLeod and Martin, 2020). In the context of the overlapping emergencies of the housing crisis, the COVID-19 pandemic and the overdose emergency declared in BC in April 2016 (Government of British Columbia, 2016), people leaving prison are at heightened risk of harm and death due to the toxic drug supply. During the pandemic, BC has seen the highest rates of overdose and overdose deaths since the public health emergency was declared (BC Centre for Disease Control, 2020). The weeks after release are already a period of high risk (Binswanger *et al.*, 2007) due to intertwined contributing factors such as interruptions in medical care, lack of needed financial resources and widespread social stigma which can impact access to housing and employment services and supports (Joudrey *et al.*, 2019). During COVID-19, people leaving prison may be at greater risk if they are unaware of changes that have resulted from responses to COVID-19, such as increased toxicity of the drug supply due to disruptions in supply chains and boarder closures (BCCDC Harm Reduction Services, 2020; Canadian Centre on Substance Use and Addiction and Canadian Community Epidemiology Network on Drug Use, 2020), or exemptions to the Controlled Drugs and Substances Act which allow for longer prescriptions and delivery of controlled substances when in quarantine or isolation (Ministry of Health, 2020).

Peer mentorship, wherein a person with lived experience of incarceration provides support to people recently released from prison, can play an important role in addressing the complex needs facing individuals released from carceral contexts. Research on peer mentorship programs in the context of the criminal legal system has demonstrated immense benefits, including improved health outcomes (South *et al.*, 2014; Cunningham *et al.*, 2018; McLeod *et al.*, 2019), feelings of belonging (Collica, 2010; Fels *et al.*, 2014; South *et al.*, 2016) and connection with employment and disability services (Hunter and Boyce, 2009; Redcross *et al.*, 2016; Harrod, 2019). For many people, release can mean feelings of stress and hopelessness (Kitson-Boyce *et al.*, 2018; Liem and Weggemans, 2018; McLeod *et al.*, 2020), peers are uniquely positioned to walk alongside people in offering comfort, wisdom and support.

The Unlocking the Gates (UTG) Peer Health Mentor program was developed by women who were previously incarcerated in BC and has been offered since 2013 (Martin *et al.*, 2009). Peer health mentors (PHM), who have themselves experienced incarceration, meet people when they are released to provide mentorship and support to access services such as housing, clothing, food, health care and social assistance. Though the formal mentorship period is three days, PHM often remain in contact with clients to provide ongoing support (McLeod *et al.*, 2020). At the time of this study, ten PHM are employed with the program. UTG works primarily with people released from BC’s provincial correctional centres, though offers support to people in correctional centres across the province (in Canada, people remanded to custody or who have been sentenced to less than two years are held in provincial/territorial correctional facilities, people sentenced to two years or more are held in federal facilities). In a 2018 study of the UTG program, 85% of participants had accessed at least one community resource within three days of release and 93% reported that their mentor assisted them to access resources. (McLeod *et al.*, 2020). PHM also offer moral, emotional and spiritual support, while meeting individual needs as they arise. Drawing on focus groups conducted with PHM, this paper reports on the experiences and perspectives of PHM who are continuing to work in their essential frontline roles during the COVID-19 and overdose public health emergencies.

## Methods

Focus group questions were collaboratively developed by KEM, KT, LLC, MK and REM rooted in commitments to critical theory, transformative justice and participatory methodologies (Mezirow, 2000; Wallerstein and Duran, 2006; Mertens, 2007; DePalma, 2010). Eight PHM participated in two focus groups held by video conference and led by KEM and KT. Focus groups were audio recorded and transcribed and all participants received transcripts for review. Each focus group participant was identified using a randomly assigned letter. Qualitative analysis was conducted using narrative thematic analysis (Riessman, 2008). One author (KEM) developed codes and initial themes from the transcripts. Co-authors and participants collaborated to iteratively revise themes. All participants received a copy of the draft manuscript for review and to provide feedback. Ethics approval for this study was granted by the University of British Columbia (#H19-00437).

## Results

Our findings highlighted the important role of PHM in supporting people leaving prison during the current health emergencies and beyond. PHM discussed opportunities for collaboration, ways the pandemic has changed how they are able to provide support and how the UTG program has remained responsive to client needs. Additionally, PHM illuminated ways that COVID-19 has exacerbated existing barriers and identified specific actions needed to support client health and rights moving forward.

### Peer support is essential

PHM viewed their work as an essential service, particularly in the context of COVID-19, when so many other resources were closed or reducing access:

We’re essential service because people are still getting out of prison. Nothing has changed […] we still want to be there for this community and we’re still passionate about helping people. (R)

I’m really grateful to be able to do the job […] But especially now in this time where some of our most vulnerable people are falling through the cracks. (J)

PHM highlighted that their own lived experience of incarceration was both a motivation to continue their work and an important part of providing meaningful support:

I did lengthy federal and provincial sentences […] when I got out a few times I had nothing to go to. And I went straight back to what I used to know and ended up right back inside. So, I find it really rewarding being able to help the guys. (A)

Because we’ve all been through that. So that’s why I think it’s really important to keep this going. It’s a very crucial time in people’s lives and very important for them to feel the warmth of inclusion from the moment they make that phone call. (J)

### Changing relationship with corrections

PHM felt that their ongoing presence during the pandemic has enhanced their relationships with provincial correctional staff. Some of the PHM reported that they have been increasingly included in collaborative and coordinated discharge planning, beginning while clients are still in custody:

They’ve been really amazing at working with us in regards to the person’s release plans. And so that they bring together all of the resources in the community that are able to help. (J)

This [release plan meeting] was amazing because the client, he led the meeting himself. […] So it was really neat to see someone taking charge of their own release plan, and it was nice to see all the organizations jump in […] So hopefully this will set precedents for more collaborations like this. (R)

These operational changes and improved relationships with correctional staff have resulted in PHM feeling more appreciated and trusted:

That door was so closed before and now, you know, staff are open […] they reach out to us […] And it just gives me so much hope that when this is over this relationship will be maintained past this pandemic. (R)

We’ve always been, you know, the ex-con group […] And I think now they really realize, like, we’re not going anywhere. We’re here. We’ve got your back. (E)

Finally, PHM report noticing more compassion from correctional staff towards individuals in custody and those planning for their release:

They’re not about punishment right now, and they’re really about care and understanding and, you know, really worried about their health. (E)

### Changes to Unlocking the Gates supports

COVID-19 has dramatically changed the support PHM can offer clients. One of the most difficult changes has been finding housing:

It’s challenging. A lot of people aren’t accepting new clients in recovery houses. Rental people are hesitant to take on new rentals because they’re scared people will move in and not pay their rent […] there was already tons of barriers with housing. But it’s become even more difficult now. (R)

In addition, PHM provide clients with supports specific to COVID-19, including sharing information about public health protocols in the community and offering masks and hand sanitizer. PHM also supply naloxone kits to any client released without one, and ensure they are trained on how to use it:

I think the mere shock of what’s happening out here for people would almost be impossible to navigate if it weren’t for somebody who was able to go through it with them and have a deeper understanding of what they need and what’s important. (J)

Uncertainty about COVID-19 has heightened anxiety around release for people in custody, who are increasingly reaching out to the UTG program for emotional support in addition to release planning:

We’ve always given […] lots of moral support. But I just find more people reaching out just for that. (R)

[They’re] grateful that we’re still here and we’re still answering our phone. And a lot of them just reach out just to talk. ‘Cause there’s so much unknown out here. And they know everything’s shut down and they’re, like, ‘what’s going to happen to me?’ (E)

PHM have also extended their support to previous clients. Some have decided to enter drug treatment because of their concerns about safety during COVID-19; the longstanding relationships they have with PHM helps to support those next steps:

I’ve had about three former clients reach out who now are interested in going to recovery because the stakes are so high. And so, I just help them because it’s– I feel like that’s the right thing to do. (J)

Previous clients have also been reaching out to check on the safety and well-being of PHM themselves, highlighting the importance of relationships built in the UTG program.

### How Unlocking the Gates has been able to continue providing services

To continue their vital work, UTG PHM introduced safety protocols to keep clients, mentors and communities safe. This has included working with corrections staff to ensure clients are symptom-free and shifting to mentoring by phone after the first day. PHM also restricted the number of stops they will make when driving with a client. When a stop is unavoidable, PHM support clients to access services safely and in alignment with public health rules:

I brought her clothing. I brought her stuff like a snack. But she really needed to be able to cash her cheque to pay if she needed a hotel room. […] I take them somewhere that I know is pretty calm. I’m with them. (B)

These protocols are important not only for client and mentor safety but also in enabling access to remaining community resources:

We’re working with a couple of treatment centres that are still taking men on the condition that they go from point A to point B with Unlocking the Gates. (E)

To maximize resources available to each of them, PHM have begun to work even more closely together to share information and support:

I knew my own area and how that worked and what’s available out here, and where I can go and how quickly I can get somebody in through a back door as opposed to the– because I know everyone. But it’s not like that anymore. So, it’s nice to have other people working on housing with you, in collaboration with you. Because it’s always good to have a few more hands and heads in the game. (J)

PHM have also drawn support from their personal networks to provide for their clients. PHM put out calls on their personal social media accounts for items such as clothing, hygiene products, masks and tents. This resourcefulness has been essential to fill gaps that exist because of widespread closures:

People are so great, because we tend to have another network of recovering addicts like us who go ‘oh, okay’, and then we start compiling these little things of clothes. (J)

PHM are able to respond flexibly to client needs while adhering to public safety guidelines by drawing on their personal networks, and support from each other, First Nation Health Authority and BC Corrections.

### Challenges

PHM identified a number of challenges exacerbated by COVID-19. First and foremost, the closure and reduction of community resources impacted their ability to meet client needs. As one peer health mentor stated, “*we were short of resources as it was, and we’re very short of resources now” (E).* Additionally, remaining community services are often more strict and rigid, impacting client outcomes. This has an important impact on the availability of housing and recovery services. Stringent requirements of those that remain open to people leaving prison has made it more difficult for PHM to find housing which meets client readiness and need:

[The UTG team] has never let anyone fall through the cracks with housing, but once somebody leaves the housing situation, to get them back into it afterwards is so tough. And I feel like that’s so heavy on my heart, because […] they’re not getting the same leeway as they did before. It’s unfortunate. (J)

The current inflexibility of community services was frustrating to PHM as well as to clients. In particular, clients who had engaged with UTG prior to COVID-19 had high expectations of the availability of support and resources in the community which could not be met under the pandemic:

They get out this time and they’re, like, I want to go here. I want to do this […] And you’ve got to explain to them, that’s COVID-19 […] The only time I’ve had problems with anybody is if they’re people that [UTG PHM] helped before. (B)

### What we need

PHM identified several priority changes needed to support the health, well-being and human rights of individuals leaving correctional facilities. The peer health mentors stressed that these needs predate COVID-19; the pandemic has highlighted existing cracks in the system and amplified the harms that result for people and communities. Housing is a persistent challenge for individuals leaving correctional facilities which has been exacerbated by closures and restrictions due to the pandemic. PHM identified the need for first stage housing so that individuals can have time to transition with support:

A place for people to go to live where these things are provided in a safe, social distancing way. So that people can work on their next plan […] We need to kind of meet people where they’re at. (J)

In addition to housing, more recovery beds would provide increased opportunity for individuals to access treatment when they feel they might be ready:

When I went to recovery,[…] I was there for all the wrong reasons. I just wanted out of jail. I didn’t want anything to do with recovery. But I stayed there and […] gradually things fell into place. So, just creating those opportunities for beds to be available for when– if people choose– they do want to give it a try. Maybe they won’t get it right the first time, maybe it’ll take two or three times or however many times. But, it’s important people have spaces to go when they do want to- if they do decide they want to give it a try. (R)

PHM discussed the importance of providing tools to support social inclusion and emotional well-being after release from prison. During the pandemic, challenges connecting to individuals and resources have intensified, especially for those without reliable access to the internet:

A lot of people that don’t have anything would go to the library and log on and they get […] free time on the internet […] you can’t even do that now. […] There’s no such thing as a payphone anymore. That’s unheard of. So, I mean, to reach out to anybody is tough. (A)

PHM saw the potential for increased supports to help people connect, or re-connect, with family and loved ones:

Now they’re worried about their children. They’re worried about their elderly parents. They’re worried about their sisters […] I really see that shift that people are really starting to worry about family, worrying about themselves. (E)

## Discussion and conclusions

UTG provides an example of the important work of peer health mentors in supporting people being released from prison, particularly in the context of the COVID-19 pandemic and the overdose emergency. PHM stressed that the pandemic is highlighting long-standing cracks in social and criminal legal systems. Finding safe, appropriate housing is a critical challenge for people leaving carceral contexts (Bowpitt and de Motte, 2019; McLeod *et al.*, 2020), which has been made even more complex during the pandemic due to the need for physical distancing, self-quarantine and reduced housing resources in the community. The calls from PHM to provide safe, stable and accessible housing to people released from prison has been echoed by others as an essential part of a public health response to COVID-19 (Franco-Paredes *et al.*, 2020; Howell *et al.*, 2020; Johnson and Beletsky, 2020). As the pandemic continues, it is critical that community services, especially housing resources, find ways to respond with more flexibility and compassion to unmet need in the community. The voices and experiences of people with unmet need and those accessing services should be central to this work.

PHM emphasized the need to provide communication tools such as phones to support people to connect with community services. Access to phones during the pandemic, especially smartphones which enable internet access, have been increasingly recognized as an essential resource to contact support services, including health care (*CBC News*, 2020; Silva, 2020). For people experiencing incarceration, access to basic communication tools should be prioritized as part of release planning (Howell *et al.*, 2020; Johnson and Beletsky, 2020).

During the pandemic, phones also play an increasingly important role in establishing and maintaining connection with loved ones both while people are incarcerated and after release. Peer mentors can support people to nurture connection and relationships in the context of COVID-19 by helping them to navigate health and safety protocols such as physical-distancing, and to use remote connection tools such as social media and video calls.

Our findings highlighted the positive impact of collaborative relationships with BC Corrections correctional and health staff in implementing coordinated release plans. This integration of provincial prisons in the public health response may be in part because health care in provincial prisons in BC was transitioned to the responsibility of the Provincial Health Services Authority, under the Ministry of Health, in 2017 (The Government of British Columbia, 2017; Pelletier *et al.*, 2018; Murdoch, 2020). BC Corrections reports that through collaboration with the Provincial Health Services Authority, corrections staff and people in custody are offered information sessions and handouts about COVID-19 transmission and safety protocols (Murdoch, 2020). Further research is needed to explore ways lessons learned from the integrated health system in BC may be applied to support correctional facilities and public health efforts around the world.

PHM accessed information and resources related to COVID-19 and the province’s public health measures from the BC Centre for Disease Control website. Having a trusted digital source allowed PHM to ensure that their work, and the guidance they give clients, kept up with changes to public health guidelines. Ensuring current information is available and accessible to people who are incarcerated, who are leaving prison, and their support systems should be a priority for correctional facilities and for public health.

The COVID-19 pandemic has exacerbated inequities and barriers faced by people in prisons. As before the pandemic, the harms of these inequities fall heaviest on people and communities disproportionately impacted by systemic injustices in the Canadian criminal legal system, including Indigenous Peoples. Some important aspects of release in BC differ from other regions, such as access to community harm-reduction supports and a universal public health-care system. Despite this, the common experiences of barriers and challenges that people face on release, and the unique benefits of peer mentorship, mean that the lessons learned from the PHM are applicable in jurisdictions across Canada and in other parts of the world. Our findings highlight the importance of peer mentorship for people leaving prison and point to the need to implement and expand peer health mentorship programs. Through their continuing work, UTG PHM provide insight into changes needed to improve release planning and to support the holistic health, well-being and human rights of people leaving prison during COVID-19.

## Acknowledgments

The authors acknowledge that this study and the UTG program were conducted across the unceded traditional territories of 198 First Nations. They also thank the peer health mentors who continue to work in this difficult time and who shared their voices and wisdom for this study. The authors honour and mourn those who have died as a result of the overdose epidemic that has swept across British Columbia. They also gratefully acknowledge funding support for the Unlocking the Gates Peer Health Mentor program from the First Nations Health Authority, and from individual philanthropic donors. The funding sponsors had no role in the design of this study; in the collection, analyses or interpretation of data; in the writing of the manuscript, or in the decision to publish the results.
